# Lower Incidence of HCC and Other Major Adverse Liver Outcomes in People Living With HIV and Chronic Liver Disease

**DOI:** 10.1016/j.gastha.2024.05.009

**Published:** 2024-06-04

**Authors:** Maurice Michel, Hannes Hagström, Linnea Widman, Piotr Nowak, Ying Shang, Jörn M. Schattenberg, Axel Wester

**Affiliations:** 1 Department of Internal Medicine II, University Medical Centre Saarland, Homburg, Germany; 2 Department of Medicine, Karolinska Institutet, Stockholm, Sweden; 3 Unit of Hepatology, Department of Upper GI Diseases, Karolinska University Hospital, Stockholm, Sweden; 4 Unit of Infectious Diseases, Karolinska University Hospital, Stockholm, Sweden; 5 Faculty of Medicine, Saarland University, Saarbrücken, Germany

**Keywords:** Chronic Liver Disease, HIV, Viral Hepatitis, Population-Based Register Study, Epidemiology

## Abstract

**Background and Aims:**

People living with human immunodeficiency virus (HIV) (PLWH) show a high incidence of chronic liver disease (CLD). However, whether HIV is associated with major adverse liver outcomes (MALO) in patients with underlying CLD remains to be determined.

**Methods:**

In this population-based cohort study, data were retrieved from the Swedish National Patient Register to identify PLWH and CLD (n = 2375) or CLD without HIV (n = 144,346) between 1997 and 2020. The cumulative incidence of MALO was calculated while accounting for competing risks (non-MALO death). Incidence rates per 1000 person-years were compared between the exposure groups (HIV vs no HIV) with Cox regression to estimate adjusted hazard ratios (HRs) and their 95% confidence intervals (CIs).

**Results:**

The incidence rate per 1000 person-years of MALO was lower in PLWH (5.1, 95% CI 4.2–6.1) compared to patients without HIV (13.1, 95% CI 12.9–13.3). This translated into an adjusted HR of 0.77 (95% CI 0.64–0.93), driven by a lower rate of hepatocellular carcinoma (adjusted HR = 0.61, 95% CI 0.43–0.86). Consistent results were noted across a range of subgroup analyses. The 10-year cumulative incidence of MALO was lower in PLWH (5.0%, 95% CI 4.1–6.1) than in patients without HIV (10.9%, 95% CI 10.7–11.0).

**Conclusion:**

Among patients with CLD, the risk of MALO was lower in PLWH compared to those without HIV, primarily due to a lower incidence of hepatocellular carcinoma. These results suggest that HIV is not associated with a higher risk of MALO.

## Introduction

Human immunodeficiency virus (HIV) is one of the most common chronic infectious diseases globally.^1^ With the advent of antiretroviral therapy in people living with HIV (PLWH), life expectancy has become comparable to the general population due to a decrease in mortality related to acquired immunodeficiency syndrome (AIDS).^2^ However, chronic liver disease (CLD) has become the most common non-AIDS–related cause of mortality in PLWH.^3^ The predominant etiologies of CLD in PLWH remain coinfections with hepatitis B virus (HBV), hepatitis C virus (HCV), or both, due to shared risk factors and transmission routes.^4^ Moreover, the prevalence of nonviral liver diseases, including metabolic dysfunction-associated steatotic liver disease (MASLD) and alcohol-associated liver disease (ALD), is increasing and these diseases have become leading indications for liver transplantation in PLWH in the United States.^5^, ^6^, ^7^ Progression of CLD can result in the development of major adverse liver outcomes (MALO), including decompensated cirrhosis and hepatocellular carcinoma (HCC), leading to increased mortality.^8^^,^^9^

An HIV infection may accelerate the progression to MALO as a result of multiple overlapping factors. The infection itself can impact liver fibrogenesis and drive steatohepatitis if no adequate viral suppression is achieved by antiretroviral therapy.^10^^,^^11^ Additionally, the high frequency of HBV and HCV coinfections in PLWH contributes to the progression to liver cirrhosis and HCC.^12^^,^^13^ A higher prevalence of metabolic risk factors and substance use disorders may further add to the burden of CLD in PLWH.^14^^,^^15^ Moreover, socioeconomic factors and regional differences in access to care for PLWH exist and may impact the development of CLD and MALO.^4^^,^^16^

Currently, there are limited data on whether HIV is associated with a different risk of MALO in patients with CLD, especially within the context of Sweden where more than 97% of diagnosed patients receive antiretroviral therapy.^17^ Therefore, the aim of this study was to examine the association between HIV and MALO in patients with underlying CLD.

## Methods

### Data Sources

In this analysis, the DEcoding the epidemiology of LIVER disease in Sweden (DELIVER) cohort was used to identify patients and outcomes.^18^ DELIVER contains data from Swedish national healthcare registers on all patients with any CLD in Sweden between 1964 and 2020 based on International Classification of Diseases (ICD) codes. The Swedish National Patient Register holds ICD codes from inpatient care since 1964 and specialized outpatient care since 2001.^19^ The positive predictive value in this register has been estimated to be 85% to 95% for most diagnoses, 91% for MASLD, and >90% for diagnoses related to cirrhosis.^19^, ^20^, ^21^ In DELIVER, the National Patient Register has been linked to several other registers, including the Cause of Death Register^22^ and the Cancer Register.^23^

### Study Population

**Figure 1:**
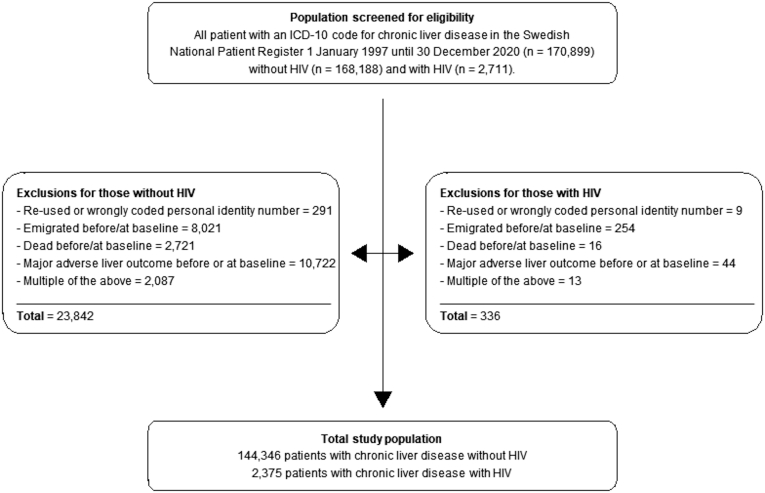
Flowchart of study exclusions.

All individuals with evidence of CLD, according to the respective ICD-10 codes for ALD, MASLD, viral hepatitis, autoimmune liver disease, cryptogenic cirrhosis, and other etiologies (ie, hemochromatosis, Wilson’s disease, Budd-Chiari syndrome, or alpha-1 antitrypsin deficiency), were identified in DELIVER (Table A1). In addition, patients with CLD were screened for HIV infection based on ICD-10 codes (Table A2). Patients were then divided into 2 groups: CLD with HIV and CLD without HIV where the first occurrence of CLD defines the baseline. Exclusion criteria included the following: re-used or wrongly coded personal identity number, emigration before or at baseline, death before or at baseline, and MALO before or at baseline. A study flow diagram of eligible patients and exclusion criteria is seen in Figure 1.

### Definition of Outcomes and Covariates

The primary outcome of interest was the first event of MALO during follow-up. MALO were defined according to the respective ICD-10 codes in the National Patient Register (main or secondary diagnosis) or the Causes of Death Register (main or contributing cause of death) as a composite of the following diagnoses: ascites, bleeding esophageal varices, hepatorenal syndrome, portal hypertension, HCC, or liver transplantation (Table A3). HCC was additionally identified in the Cancer Register. The individual components of MALO were considered secondary outcomes. The end of follow-up was defined as the date of an outcome, emigration from Sweden, end of the study period (December 31, 2020), or death unrelated to the outcome of interest. Patients without HIV at baseline were censored if they received a diagnosis of HIV during follow-up, and then entered the study in the PLWH group instead. Covariates included comorbidities at or before baseline in the National Patient Register (main or secondary diagnosis), and they were defined according to the respective ICD-10 codes (Table A4): cardiovascular disease (CVD), hypertension, hyperlipidemia, type 2 diabetes, obesity, cancer, chronic obstructive pulmonary disease, mental health disorders, substance use disorder, chronic kidney disease, and compensated cirrhosis.

### Subgroups

The primary outcome was further analyzed in subgroups according to sex, different age groups (<50, 50–65, >65), year of inclusion (1997–2004, 2005–2013, 2014–2020), and liver disease etiology (ALD and viral hepatitis, ALD without viral hepatitis, viral hepatitis without ALD, MASLD, autoimmune liver disease, other, and cryptogenic cirrhosis). Furthermore, subgroups were also formed for liver disease severity (compensated cirrhosis or no cirrhosis at baseline) and whether metabolic risk factors were present or not at baseline (hypertension, hyperlipidemia, type 2 diabetes, or obesity).

### Statistical Analysis

Baseline characteristics were compared either using Wilcoxon rank-sum or Pearson’s chi-squared test to calculate *P* values. A *P* value <.05 was considered statistically significant. Incidence rates per 1000 person-years (PY) of the primary and secondary outcomes were calculated. Cox regression models were fitted to estimate unadjusted and adjusted hazard ratios (aHRs) and their 95% confidence intervals (CIs). Adjustments were made as follows: age, sex, inclusion year, education (<10, 10–12, >12 years), country of birth (Nordic country or other), CVD, hypertension, hyperlipidemia, type 2 diabetes, obesity, cancer, chronic obstructive pulmonary disease (as a proxy for cigarette smoking), chronic kidney disease, compensated cirrhosis, and liver disease etiology. Adjusting for compensated cirrhosis and liver disease etiology had the intention to capture the impact of these conditions on MALO. In addition, we calculated the E-value for the primary outcome (MALO).^24^ The cumulative incidence of the primary outcome (MALO) at 1, 5, and 10 years after baseline and at the full follow-up was calculated using the Aalen-Johansen estimator while accounting for the competing risk of non-MALO death.

Furthermore, we performed sensitivity analyses of the primary outcome. First, hepatorenal syndrome and portal hypertension were excluded from the MALO definition to detect any difference in the primary outcome. Second, adjustments were also made for mental health disorders and substance use disorders since these comorbidities may affect attending surveillance and compliance with treatment.^16^^,^^25^ Third, we excluded patients who had received direct-acting antiviral (DAA) treatment before or at baseline and censored patients if they filled a prescription for DAA during follow-up. All analyses were executed using Stata, version 16.1 (Stata-Corp, College Station, TX).

## Results

### Baseline Characteristics

A total of 146,721 patients with CLD were included in the study, of whom 2375 were PLWH and 144,346 were patients without HIV. Baseline characteristics of included patients are shown in Table 1. Most patients were male in both groups, and the median age at baseline was 41 years in PLWH and 49 years in patients without HIV. In both groups, the main etiology of CLD was viral hepatitis without ALD, with a higher percentage in PLWH than in patients without HIV (91.7% vs 50.5%). HCV (67.9%) was the predominant cause of CLD in PLWH followed by HBV (23.9%). Other common etiologies of CLD were ALD without viral hepatitis, and MASLD, especially in patients without HIV. Liver disease severity in terms of compensated cirrhosis was lower in PLWH at baseline (2.9% vs no HIV: 14.9%, *P* < .001). CVD and metabolic risk factors were more prevalent in patients without HIV. Common comorbidities in PLWH were mental health (24.7%) and substance use disorders (45.6%).

**Table 1:** Baseline Characteristics

Variable	Patients with chronic liver disease and HIV	Patients with chronic liver disease without HIV	*P* value
Included persons, n	2375	144,346	
Follow-up (y) (median, range, IQR)	9.0 (4.5–13.2)	6.9 (2.6–13.1)	<.001
Person-years of follow-up	21,728	1,186,896	
Sex, men, n (%)	1632 (68.7)	84,563 (58.6)	<.001
Age at baseline, y (median, IQR)	41 (33–49)	49 (35–63)	<.001
Period of inclusion, n (%)			< .001
1997–2004	596 (25.1)	44,877 (31.1)	
2005–2013	1263 (53.2)	54,481 (37.7)	
2014–2020	516 (21.7)	44,988 (31.2)	
Country of birth, n (%)			<.001
Nordic	1622 (68.3)	110,393 (76.5)	
Other	753 (31.7)	33,891 (23.5)	
Unknown	0 (0.0)	62 (0.0)	
Education, n (%)			.005
<10 y	835 (35.2)	46,404 (32.1)	
10–12 y	1037 (43.7)	63,535 (44.0)	
>12 y	446 (18.8)	29,912 (20.7)	
Missing	57 (2.4)	4495 (3.1)	
Liver disease etiology, n (%)			<.001
ALD + viral hepatitis	15 (0.6)	397 (0.3)	
ALD without viral hepatitis	62 (2.6)	19,820 (13.7)	
Viral hepatitis without ALD	2179 (91.7)	72,897 (50.5)	
HCV without HBV and without ALD	1612 (67.9)	48,805 (33.8)	
HBV without ALD^a^	567 (23.9)	24,092 (16.7)	
MASLD	58 (2.4)	16,738 (11.6)	
Autoimmune liver disease	26 (1.1)	12,734 (8.8)	
Other liver disease	13 (0.5)	12,427 (8.6)	
Cryptogenic cirrhosis	22 (0.9)	9333 (6.5)	
Liver disease severity, n (%)			<.001
Cirrhosis	70 (2.9)	21,472 (14.9)	
No cirrhosis	2305 (97.1)	122,874 (85.1)	
Comorbidity at or before baseline, n (%)			
CVD	131 (5.5)	16,663 (11.5)	<.001
Metabolic risk factor	223 (9.4)	31,676 (21.9)	
Hypertension	135 (5.7)	22,682 (15.7)	<.001
Hyperlipidemia	64 (2.7)	6490 (4.5)	<.001
Type 2 diabetes	72 (3.0)	12,439 (8.6)	<.001
Obesity	29 (1.2)	5594 (3.9)	<.001
Non-HCC cancer	79 (3.3)	10,946 (7.6)	<.001
COPD	52 (2.2)	5509 (3.8)	<.001
Mental health disorder	586 (24.7)	22,591 (15.7)	<.001
Substance use disorder	1083 (45.6)	36,144 (25.0)	<.001
Chronic kidney disease	19 (0.8)	2237 (1.5)	.003

Wilcoxon rank-sum or Pearson’s chi-squared test were used to compare groups and calculate *P* values. A *P* value <.05 was considered statistically significant.

ALD, alcohol-associated liver disease; COPD, chronic obstructive pulmonary disease; CVD, cardiovascular disease; HBV, hepatitis B virus; HCV, hepatitis C virus; HCC, hepatocellular carcinoma; HIV, human immunodeficiency virus; IQR, interquartile range; MASLD, metabolic dysfunction-associated steatotic liver disease.

a Those coding for both HBV and HCV fall into the group with HBV.

### Rate of MALO and Its Components

The incidence rate (95% CI) of MALO was lower in PLWH (5.1/1000 PY, 95% CI 4.2–6.1) compared to patients without HIV (13.1/1000 PY, 95% CI 12.9–13.3). The rate of MALO was also lower in PLWH after multivariable adjustments, including adjustments for compensated cirrhosis and liver disease etiology (aHR = 0.77, 95% CI 0.64–0.93). After excluding hepatorenal syndrome and portal hypertension from the MALO definition, a similar lower rate of MALO in PLWH compared to patients without HIV was found (aHR = 0.74, 95% CI 0.61–0.91). The E-value for MALO was 1.92 and 1.36 for the corresponding CI. The incidence rate of the individual components of MALO were all lower in PLWH compared to patients without HIV, including a lower incidence rate of HCC (1.5/1000 PY, 95% CI 1.1–2.1 vs no HIV: 3.4/1000 PY, 95% CI 3.3–3.5). The rate of HCC was lower in PLWH than in patients without HIV (aHR = 0.61, 95% CI 0.43–0.86) (Table 2). The rate of MALO remained lower in PLWH for DAA-naïve patients (aHR = 0.79, 95% CI 0.64–0.97) and after additional adjustments for mental health and substance use disorders (aHR = 0.77, 95% CI 0.64–0.94).

**Table 2:** Incident Rate and Hazard Ratio of Major Adverse Liver Outcomes and Its Individual Components

Outcome	Events, patients with HIV, n (%)	Events, patients without HIV, n (%)	Incidence rate/1000 PY (95% CI), patients with HIV	Incidence rate/1000 PY (95% CI), patients without HIV	Unadjusted HR (95% CI)	Adjusted HR (95% CI)
Major adverse liver outcomes	110 (4.6)	15,506 (10.7)	5.1 (4.2–6.1)	13.1 (12.9–13.3)	0.40 (0.33–0.48)	0.77 (0.64–0.93)
Ascites	56 (2.4)	8305 (5.8)	2.5 (2.0–3.3)	6.8 (6.7–7.0)	0.38 (0.29–0.49)	0.83 (0.64–1.08)
Bleeding varices	25 (1.1)	3961 (2.7)	1.1 (0.8–1.7)	3.2 (3.1–3.3)	0.35 (0.24–0.52)	0.76 (0.51–1.13)
Hepatorenal syndrome	5 (0.2)	1560 (1.1)	0.2 (0.1–0.5)	1.3 (1.2–1.3)	0.18 (0.08–0.43)	0.45 (0.19–1.09)
Portal hypertension	28 (1.2)	3117 (2.2)	1.3 (0.9–1.8)	2.5 (2.4–2.6)	0.52 (0.36–0.76)	1.11 (0.76–1.61)
HCC	33 (1.4)	4135 (2.9)	1.5 (1.1–2.1)	3.4 (3.3–3.5)	0.45 (0.32–0.64)	0.61 (0.43–0.86)
Liver transplantation	14 (0.6)	1932 (1.3)	0.6 (0.4–1.1)	1.6 (1.5–1.7)	0.40 (0.24–0.68)	0.68 (0.40–1.15)

Adjustments: age, sex, inclusion year, education (<10, 10–12, >12 y), country of birth (Nordic country or other), CVD, hypertension, hyperlipidemia, type 2 diabetes, obesity, cancer, COPD, chronic kidney disease, compensated cirrhosis, and liver disease etiology.

CI, confidence interval; COPD, chronic obstructive pulmonary disease; CVD, cardiovascular disease; HCC, hepatocellular carcinoma; HR, hazard ratio; HIV, human immunodeficiency virus; PY, person-years.

### Rate of MALO in Subgroups

Table 3 presents the rate of MALO in subgroups. The rate of MALO was lower in PLWH across all subgroups, although many estimates were imprecise. The rate was reduced in PLWH for both men (aHR = 0.79, 95% CI 0.64–0.98) and women (aHR = 0.65, 95% CI 0.41–1.02), and across all age groups and inclusion years compared to patients without HIV. Consistent results were found for all liver disease etiologies. The rate of MALO was lower in PLWH compared to patients without HIV both in patients with compensated cirrhosis (aHR = 0.70, 95% CI 0.46–1.08) and in patients without cirrhosis at baseline (aHR = 0.79, 95% CI 0.64–0.98). Moreover, PLWH had a lower rate of MALO regardless of whether metabolic risk factors were present (aHR = 0.64, 95% CI 0.39–1.06) or not (aHR = 0.80, 95% CI 0.65–0.98). The rate of HCC was lower in PLWH for both patients with (aHR = 0.43, 95% CI 0.14–1.33) and without cirrhosis at baseline (aHR = 0.65, 95% CI 0.45–0.93), although estimates again were imprecise (Table A5).

**Table 3:** Incident Rate and Hazard Ratio of Major Adverse Liver Outcomes in Subgroups

Subgroup	Events, patients with HIV, n (%)	Events, patients without HIV, n (%)	Incidence rate/1000 PY (95% CI), patients with HIV	Incidence rate/1000 PY (95% CI), patients without HIV	Unadjusted HR (95% CI)	Adjusted HR (95% CI)
Sex						
Men	91 (5.6)	10,284 (12.2)	6.3 (5.1–7.7)	15.2 (14.9–15.5)	0.42 (0.34–0.52)	0.79 (0.64–0.98)
Women	19 (2.6)	5222 (8.7)	2.6 (1.7–4.1)	10.2 (10.0–10.5)	0.27 (0.17–0.42)	0.65 (0.41–1.02)
Age group						
<50	57 (3.2)	3874 (5.4)	3.2 (2.5–4.1)	5.2 (5.1–5.4)	0.61 (0.47–0.80)	0.88 (0.67–1.14)
50–65	47 (9.3)	6931 (16.3)	13.2 (9.9–17.5)	21.6 (21.1–22.1)	0.58 (0.44–0.77)	0.72 (0.54–0.96)
>65	6 (9.7)	4701 (15.9)	18.4 (8.3–41.0)	36.9 (35.8–37.9)	0.53 (0.24–1.17)	0.88 (0.39–1.95)
Inclusion year						
1997–2004	44 (7.4)	6452 (14.4)	5.7 (4.3–7.7)	11.2 (11.0–11.5)	0.51 (0.38–0.68)	0.86 (0.64–1.15)
2005–2013	55 (4.4)	5849 (10.7)	4.5 (3.5–5.9)	12.3 (12.0–12.7)	0.38 (0.29–0.49)	0.75 (0.58–0.98)
2014–2020	11 (2.1)	3205 (7.1)	5.8 (3.2–10.4)	23.1 (22.3–23.9)	0.27 (0.15–0.49)	0.70 (0.39–1.27)
Liver disease etiology						
ALD + viral hepatitis	2 (13.3)	166 (41.8)	17.5 (4.4–70.1)	86.1 (73.9–100.2)	0.23 (0.06–0.95)	0.34 (0.08–1.37)
ALD without viral hepatitis	9 (14.5)	5019 (25.3)	26.8 (13.9–51.5)	50.0 (48.6–51.4)	0.52 (0.27–0.997)	0.54 (0.28–1.04)
Viral hepatitis without ALD	89 (4.1)	4098 (5.6)	4.3 (3.5–5.3)	5.5 (5.4–5.7)	0.78 (0.63–0.97)	0.92 (0.75–1.14)
HCV without HBV and without ALD	72 (4.5)	3529 (7.2)	4.6 (3.7–5.8)	6.9 (6.7–7.2)	0.67 (0.53–0.84)	0.82 (0.65–1.04)
MASLD	3 (5.2)	1571 (9.4)	9.0 (2.9–27.9)	15.2 (14.4–15.9)	0.56 (0.18–1.74)	0.60 (0.19–1.89)
Autoimmune liver disease	1 (3.8)	1567 (12.3)	6.3 (0.9–44.6)	16.0 (15.2–16.8)	0.39 (0.06–2.79)	0.51 (0.07–3.64)
Other liver disease	0 (0.0)	381 (3.1)	0	3.7 (3.4–4.1)	–	–
Cryptogenic cirrhosis	6 (27.3)	2704 (29.0)	51.7 (23.2–115.0)	67.2 (64.7–69.8)	0.84 (0.38–1.87)	0.84 (0.38–1.87)
Liver disease severity						
Cirrhosis	21 (30.0)	7094 (33.0)	65.2 (42.5–100.0)	89.2 (87.2–91.3)	0.79 (0.52–1.22)	0.70 (0.46–1.08)
No cirrhosis	89 (3.9)	8412 (6.8)	4.2 (3.4–5.1)	7.6 (7.4–7.8)	0.55 (0.45–0.68)	0.79 (0.64–0.98)
Metabolic risk factor						
Yes	16 (7.2)	4582 (14.5)	11.7 (7.2–19.1)	28.7 (27.9–29.5)	0.43 (0.27–0.71)	0.64 (0.39–1.06)
No	94 (4.4)	10,924 (9.7)	4.6 (3.8–5.7)	10.6 (10.4–10.8)	0.43 (0.35–0.53)	0.80 (0.65–0.98)

Adjustments: age, sex, inclusion year, education (<10, 10–12, >12 y), country of birth (Nordic country or other), CVD, hypertension, hyperlipidemia, type 2 diabetes, obesity, cancer, COPD, chronic kidney disease, compensated cirrhosis, and liver disease etiology.

For all subgroups, patients with the subgroup characteristic are compared to controls with the same characteristic (eg, chronic liver disease + HIV + MASLD are compared to chronic liver disease without HIV + MASLD).

ALD, alcohol-associated liver disease; CI, confidence interval; HBV, hepatitis B virus; HCV, hepatitis C virus; HIV, human immunodeficiency virus; HR, hazard ratio; MASLD, metabolic dysfunction-associated steatotic liver disease; PY, person-years.

### Cumulative Incidence of MALO

**Figure 2:**
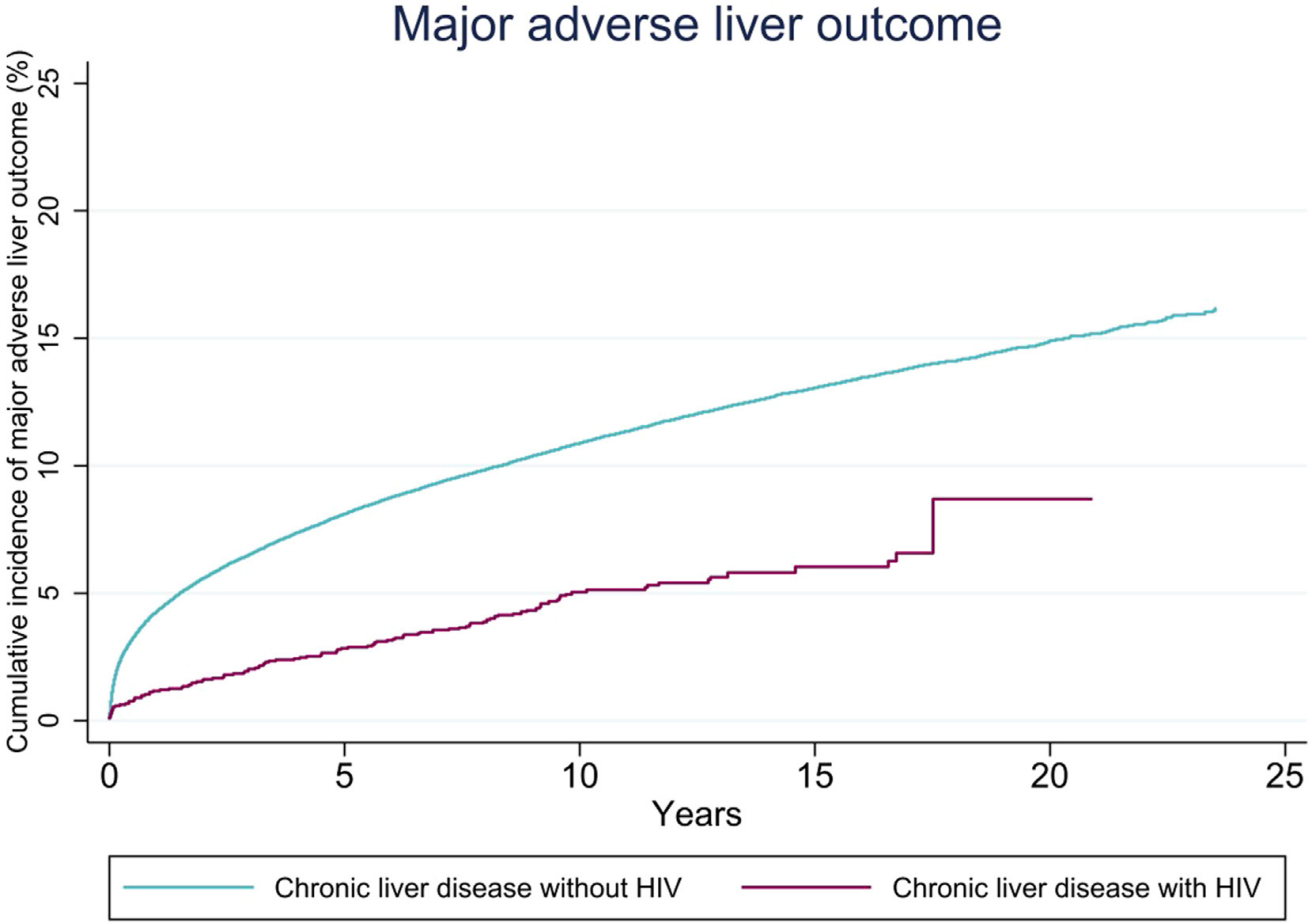
Cumulative incidence of major adverse liver outcomes in patients with chronic liver disease with and without HIV.

Considering the full follow-up period of up to 24 years, 8.7% (95% CI 5.2–13.7) of PLWH had experienced MALO compared to 16.2% (95% CI 15.7–16.6) of patients without HIV (Table 4). The cumulative incidences of MALO at 10 years of follow-up in PLWH and patients without HIV were 5.0% (95% CI 4.1–6.1) and 10.9% (95% CI 10.7–11.0), respectively. Figure 2 shows the cumulative incidence function curve of MALO in PLWH and patients without HIV. The cumulative incidences of MALO at full follow-up were lower in PLWH compared to patients without HIV across all liver disease etiologies, for both cirrhotic and noncirrhotic patients, and for both patients with and without metabolic risk factors.

**Table 4:** Cumulative Incidence of Major Adverse Liver Outcomes

Subgroup	1 y, patients with HIV (95% CI)	1 y, patients without HIV (95% CI)	5 y, patients with HIV (95% CI)	5 y, patients without HIV (95% CI)	10 y, patients with HIV (95% CI)	10 y, patients without HIV (95% CI)	Full follow-up, patients with HIV (95% CI)	Full follow-up, patients without HIV (95% CI)
Overall	1.1 (0.7–1.6)	4.2 (4.1–4.4)	2.8 (2.1–3.5)	8.1 (7.9–8.2)	5.0 (4.1–6.1)	10.9 (10.7–11.0)	8.7 (5.2–13.7)	16.2 (15.7–16.6)
Sex								
Men	1.4 (0.9–2.0)	4.8 (4.7–5.0)	3.3 (2.4–4.2)	9.2 (9.0–9.4)	6.1 (4.9–7.5)	12.3 (12.0–12.5)	11.2 (6.2–18.4)	18.0 (17.4–18.6)
Women	0.7 (0.3–1.5)	3.4 (3.3–3.6)	1.9 (1.1–3.1)	6.5 (6.3–6.7)	2.8 (1.7–4.2)	8.9 (8.6–9.1)	3.6 (1.9–6.0)	13.5 (12.8–14.1)
Age group								
<50	0.7 (0.4–1.2)	1.3 (1.2–1.4)	1.6 (1.1–2.3)	2.9 (2.8–3.0)	3.2 (2.4–4.2)	4.7 (4.5–4.9)	6.9 (3.3–12.8)	10.5 (9.9–11.1)
50–65	2.0 (1.0–3.6)	6.3 (6.0–6.5)	6.3 (4.3–8.8)	12.2 (11.9–12.6)	11.7 (8.6–15.3)	16.8 (16.4–17.2)	15.6 (9.9–22.3)	23.6 (22.6–24.6)
>65	6.5 (2.1–14.4)	8.6 (8.3–8.9)	–	15.1 (14.6–15.5)	–	18.0 (17.5–18.5)	10.3 (4.2–19.7)	19.8 (19.2–20.4)
Inclusion year								
1997–2004	1.3 (0.6–2.5)	4.1 (3.9–4.3)	3.6 (2.3–5.3)	8.1 (7.8–8.3)	6.0 (4.2–8.1)	10.9 (10.6–11.2)	9.4 (5.8–14.2)	16.3 (15.8–16.8)
2005–2013	0.8 (0.4–1.4)	4.0 (3.8–4.1)	2.4 (1.7–3.7)	7.7 (7.5–7.9)	4.6 (3.5–5.9)	10.4 (10.2–10.7)	5.0 (3.8–6.5)	12.9 (12.3–13.5)
2014–2020	2.0 (1.0–3.5)	4.8 (4.6–5.0)	–	8.5 (8.2–8.8)	–	–	2.3 (1.2–3.9)	9.6 (9.2–10.1)
Liver disease etiology								
ALD + viral hepatitis	7.2 (0.5–27.8)	20.2 (16.4–24.3)	–	34.7 (30.0–39.5)	–	40.7 (35.7–45.7)	14.4 (2.4–36.9)	47.0 (41.4–52.5)
ALD without viral hepatitis	8.1 (3.0–16.5)	13.3 (12.8–13.8)	12.3 (5.3–22.4)	22.7 (22.1–23.4)	–	26.9 (26.3–27.6)	19.6 (9.0–33.1)	30.0 (29.2–30.8)
Viral hepatitis without ALD	0.7 (0.4–1.2)	0.9 (0.9–1.0)	2.1 (1.6–2.8)	2.7 (2.6–2.8)	4.3 (3.5–5.4)	4.9 (4.7–5.0)	8.1 (4.6–13.3)	10.2 (9.6–10.7)
MASLD	3.5 (0.6–10.7)	4.3 (4.0–4.6)	–	8.7 (8.2–9.2)	–	11.6 (11.0–12.2)	6.3 (1.6–16.0)	14.9 (13.3–16.8)
Autoimmune liver disease	9.6 (0.6–34.6)	2.9 (2.6–3.2)	9.6 (0.6–34.6)	7.3 (6.8–7.7)	–	12.3 (11.6–13.0)	9.6 (0.6–34.6)	26.0 (23.0–29.2)
Other liver disease	–	0.8 (0.6–0.9)	–	1.9 (1.6–2.1)	–	3.2 (2.9–3.6)	–	6.4 (5.3–7.7)
Cryptogenic cirrhosis	18.4 (5.7–36.7)	16.9 (16.1–17.6)	–	26.8 (25.9–27.7)	–	30.9 (29.9–31.9)	30.4 (12.0–51.2)	35.1 (33.7–36.6)
Liver disease severity								
Cirrhosis	15.9 (8.5–25.4)	19.2 (18.6–19.7)	32.3 (20.9–44.3)	31.3 (30.6–31.9)	–	35.9 (35.2–36.6)	34.5 (22.6–46.7)	39.4 (38.6–40.3)
No cirrhosis	0.7 (0.4–1.1)	1.7 (1.6–1.7)	1.9 (1.4–2.6)	4.1 (4.0–4.2)	4.2 (3.3–5.2)	6.6 (6.5–6.8)	8.0 (4.5–13.4)	12.2 (11.7–12.7)
Metabolic risk factor								
Yes	2.3 (0.9–4.9)	7.0 (6.7–7.3)	5.7 (3.0–9.7)	13.3 (12.9–13.7)	–	17.0 (16.5–17.5)	10.1 (5.9–15.7)	21.2 (20.3–22.2)
No	1.0 (0.6–1.5)	3.5 (3.4–3.6)	2.5 (1.9–3.2)	6.7 (6.6–6.9)	4.6 (3.7–5.7)	9.3 (9.2–9.5)	8.4 (4.9–13.6)	15.0 (14.5–15.5)

ALD, alcohol-associated liver disease; CI, confidence interval; HIV, human immunodeficiency virus; MASLD, metabolic dysfunction-associated steatotic liver disease.

## Discussion

In this large nationwide population-based cohort study, we compared the risk of MALO in patients with CLD and HIV to patients with CLD and no HIV. The main finding was that PLWH showed an overall lower risk of MALO after adjustments for important confounding factors. This was mainly due to a lower incidence of HCC. Moreover, the severity of liver disease was reduced in PLWH as suggested by the lower prevalence of compensated cirrhosis at baseline. Collectively, our results suggest that HIV does not increase the risk of MALO in patients with CLD.

The risk of MALO in CLD was overall lower in PLWH compared to patients without HIV, mainly due to a lower incidence of HCC. This is contrary to recent analyses from populations in North America showing a higher rate of HCC in PLWH, especially if viral hepatitis is present.^26^ Viral hepatitis is the predominant liver-related comorbidity in PLWH, as confirmed by our and other studies.^3^ Coinfection with HBV and HCV is a major risk factor for the development of HCC.^8^^,^^26^ Despite the high burden of viral hepatitis in PLWH, advances in the treatment of HCV with DAA and vaccinations against HBV have improved liver-related mortality in these patients. Additionally, first-line antiretroviral therapy regimens containing tenofovir were associated with lower HCC incidence in HBV-coinfected and HCV-coinfected PLWH.^27^ Previous studies have shown that HIV coinfection in patients with HCV was not associated with a higher risk of liver-related death and HCC compared to HCV mono-infection, especially if sustained virological response was achieved.^28^, ^29^, ^30^ Additionally, other studies have found that treatment of viral coinfections in PLWH can decrease the rate of MALO.^31^ Despite treatment with antiretroviral therapy, mortality of HCC is still considered higher in PLWH independent of disease severity compared to patients without HIV.^12^ The predominant etiology of viral hepatitis was HCV in our study which is in line with previous data as Sweden is considered a low-endemic country for HBV.^32^ This could also have impacted our findings as HBV infection is associated with the highest risk of HCC, even without underlying cirrhosis.^8^ Moreover, since antiviral therapy of HCV may have resulted in fibrosis regression with less risk of MALO, this could have also resulted in a lower number of MALO compared to HIV-negative patients who were less affected by HCV in our study. The incidence of MALO and HCC was not increased in PLWH regardless of cirrhosis status at baseline. The improved treatment options for both HBV and HCV may have resulted in a slower progression of liver disease in PLWH likely contributing to a lower MALO risk in our study.

Liver disease severity at baseline was different between the investigated groups in this study. More patients without HIV had liver cirrhosis at baseline than PLWH, which could have affected our results and partly explain the lower risk of MALO in PLWH. Although we adjusted for cirrhosis status, we could not fully capture different factors determining liver disease severity besides cirrhosis in this study, such as liver fibrosis stage or liver stiffness that can impact the development of MALO, especially in PLWH and HCV coinfection.^33^^,^^34^ Besides viral hepatitis, ALD and MASLD were less prevalent in PLWH compared to HIV-negative patients despite the increasing burden in PLWH.^5^ Especially the number of MASLD in PLWH was lower compared to other recently published studies, which may be a result of a larger number of patients included from 1997 to 2013, when the entity MASLD was less relevant and familiar.^14^ Moreover, PLWH showed less metabolic risk factors, including type 2 diabetes, which are known to cause fibrosis progression.^35^ Overall, the prevalence of ALD, MASLD, and metabolic risk factors were less relevant in PLWH in our study, which could have also resulted in a lower MALO rate in PLWH.

While previous studies report a high incidence of liver-related events and HCC in PLWH even under antiretroviral therapy, the lower risk of MALO that we observed in PLWH may also be attributed to the close surveillance of these patients in Sweden.^26^^,^^36^ Suppression of HIV viremia can delay the onset of cirrhosis in HCV-coinfected patients, and mitigate the overall risk of HCC in HBV-coinfected or HCV-coinfected patients.^37^^,^^38^ Importantly, 99% of PLWH in Sweden are registered in the healthcare system and 97% receive antiretroviral therapy as part of the Swedish Communicable Disease Act with regular follow-up visits and treatment of HIV free of charge.^17^^,^^39^ In this context, Sweden was the first country to achieve the UNAIDS/WHO 90-90-90 goals. Thus, PLWH in Sweden may benefit from closer monitoring than those without HIV that could have led to the management of comorbidities earlier, possibly preventing the occurrence of MALO. That may introduce potential confounding that we could not adjust for. However, access to care and continued monitoring of treatment in PLWH remains challenging.^4^ Multiple factors, including socioeconomic status, substance use disorder, or alcohol abuse, as well as a higher prevalence of mental health disorders can limit access to care with a potential impact on the liver-related disease burden.^16^^,^^25^ In our cohort, the risk of MALO was lower in PLWH compared to patients without HIV, also after adjustment for these confounders. These results may indicate that the close surveillance and improved treatment options of PLWH may have affected liver-related outcomes.

### Strengths and Limitations

The major strength of this study is the large sample size of patients with CLD from the validated population-based Swedish National Patient Register.^19^ In contrast to other studies, we included all patients with diagnosed CLD. This approach allows for higher generalizability and minimizes selection bias commonly seen in monocentric studies. Moreover, the availability of data on comorbidities and demographic variables allowed for the adjustment of important confounders and therefore improved the internal validity of our results.

Certain limitations need to be addressed for the interpretation of our results. Liver disease severity was only assessed with the presence of compensated cirrhosis at baseline, with however no difference according to, for example, Model for End-stage Liver Disease score or the stage of fibrosis. Moreover, PLWH were younger compared to HIV-negative patients which could have also resulted in less severe liver disease, since age is known to cause fibrosis progression. No data were available on the immune status of PLWH in our study, and therefore, no estimate on the disease stage can be reported. Adherence to antiretroviral therapy was not captured; however, based on the HIV treatment outcome data, this is likely to be high.^39^ Although we captured treatment with DAA in those with HCV, we had no data if patients achieved sustained viral response. Despite the multiple subgroup analyses, we noted few events in many subgroups resulting in imprecise risk estimates, meaning that the subgroup analyses should be interpreted with caution. Unmeasured confounding could be another possible explanation for our findings that PLWH had a lower MALO rate. However, as represented in the E-value, unmeasured confounders are unlikely to have shifted the estimates to the extent that HIV would be associated with higher risk of MALO. Overall, ICD codes can lead to mislabeling of some patients. However, this is unlikely to impact the results since this limitation should affect both groups equally.

## Conclusions

In patients with CLD, the risk of MALO was lower in PLWH compared to those without HIV, which was mainly due to a lower incidence of HCC. These results indicate that HIV is not associated with a higher risk of developing MALO in patients with CLD.

## Supplementary Materials

Tables A1–A5
