# Supplementary material for: Lower Incidence of HCC and Other Major Adverse Liver Outcomes in People Living With HIV and Chronic Liver Disease
Source: Gastro Hep Adv. 2024 Jun 4;3(6):783–92. doi: 10.1016/j.gastha.2024.05.009 (PMC11401543; doi:10.1016/j.gastha.2024.05.009)
Supplement: Tables A1–A5 [file mmc1.docx]

**Supplementary Table 1: ICD-codes used to define chronic liver disease. Main or secondary diagnoses from the National Patient Register.**

|  | **ICD10** |
| --- | --- |
| **ALD** | K70 |
| **MASLD** | K76.0, K75.8 without coding for any other liver disease in this table except cryptogenic cirrhosis.  Reclassify patients with MASLD to the ALD group if they have a code for alcohol use disorder (F10) at or before the coding for MASLD without coding for any other liver disease in this table except cryptogenic cirrhosis. |
| **Viral hepatitis** | B16, B17, B18, B19 |
| **Autoimmune liver disease** | Primary sclerosing cholangitis: ([K50 or K51) + K83.0]) or K83.0A  Primary biliary cholangitis: K74.3, K74.5  Autoimmune hepatitis: K75.4 |
| **Other liver disease** | Alpha-1 antitrypsin deficiency: E88.0A, E88.0B  Budd-Chiari: I82.0, K765  Hemochromatosis: E83.1  Wilson’s disease: E83.0B |
| **Cryptogenic cirrhosis** | Unspecified cirrhosis: K74.6  Non-bleeding esophageal varices: I85.9, I98.2  Patients will only be classified into this group if they lack coding for specific liver diseases (all other codes in the table)  Reclassify patients with cryptogenic cirrhosis to the ALD group if they have a code for alcohol use disorder (F10) at or before the coding for cryptogenic cirrhosis  Reclassify patients with cryptogenic cirrhosis to the MASLD group if they have a code for type 2 diabetes (E11) at or before the coding for cryptogenic cirrhosis |

ICD, International Classification of Disease; ALD, alcohol-associated liver disease; MASLD, metabolic dysfunction-associated steatotic liver disease

**Supplementary Table 2: ICD codes used to define HIV. Main or secondary diagnosis from the National Patient Register.**

|  | **ICD10** |
| --- | --- |
| **HIV** | B24  O98.7  R75  Z11.4  Z21.9  Z71.7  B20-B23  F02.4 |

ICD, International Classification of Disease; HIV, human immunodeficiency virus.

**Supplementary Table 3: ICD codes used to define MALO. Main or secondary diagnosis from the National Patient Register and main or contributing causes of death from the Cause of Death Register. HCC was also captured from the Cancer Register.**

|  | **ICD10** |
| --- | --- |
| **Ascites** | R18 without any code for heart failure (I50) or non-HCC cancer (any C, except C22.0) at the same visit |
| **Bleeding varices** | I85.0, I98.3 |
| **Hepatorenal syndrome** | K76.7 |
| **Portal hypertension** | K76.6 |
| **HCC** | C22.0 |
| **Liver transplantation** | Diagnostic code: Z94.4  Procedure codes: JJC00, JJC10, JJC20, DJ005, DJ006, JJC30, JJC40 |

ICD, International Classification of Disease; HCC, hepatocellular carcinoma.

**Supplementary Table 4: ICD-codes for baseline comorbidities and cirrhosis. Main or secondary diagnoses from the National Patient Register.**

| **Comorbidities** | **ICD10** |
| --- | --- |
| CVD | Ischemic heart disease: I20-I25  Cerebrovascular disease: I60-I69  Heart failure: I50  Peripheral artery disease: I739  Arterial thrombosis: I74 |
| Hypertension | I10-I15 |
| Hyperlipidemia | E78 |
| Type 2 diabetes | E11 |
| Obesity | E65, E66 |
| Cancer | C (any C, except C22.0, it is part of the primary outcome and is an exclusion criterion) |
| COPD, proxy for heavy smoking | J41-J44 + age≥40 years at the time of coding |
| Mental health disorders | F20-F29, F30-F39, F40-F48 |
| Substance use disorder | F10-F19 |
| Chronic kidney disease | N18 |
| Cirrhosis | I85.9, I98.2, K70.3, K74.6, K74.5, B18.0E/G, B18.1E/G, B18.2E/G, B18.8E/G, B18.9E/G |

ICD, International Classification of Disease; CVD, cardiovascular disease; COPD, chronic obstructive pulmonary disease.

**Supplementary Table 5: Incidence rate and hazard ratio of HCC according to the presence of cirrhosis.**

|  | **Events, patients with HIV n(%)** | **Events, patients without HIV n(%)** | **Incidence rate/1000 PY (95% CI), patients with HIV** | **Incidence rate/1000 PY (95% CI), patients without HIV** | **Unadjusted HR (95% CI)** | **Adjusted HR (95% CI)** |
| --- | --- | --- | --- | --- | --- | --- |
| **HCC** |  |  |  |  |  |  |
| Cirrhosis | 3 (4.3) | 1,445 (6.7) | 7.1 (2.3-22.0) | 14.9 (14.2-15.7) | 0.51 (0.17-1.60) | 0.43 (0.14-1.33) |
| No cirrhosis | 30 (1.3) | 2,690 (2.2) | 1.4 (1.0-2.0) | 2.4 (2.3-2.5) | 0.60 (0.42-0.85) | 0.65 (0.45-0.93) |

HIV, human immunodeficiency virus; PY, person-years; CI, confidence interval; HR, hazard ratio; HCC, hepatocellular carcinoma.

Adjustment: adjusted for age, sex, inclusion year, education (<10, 10-12, >12), country of birth (Nordic country or other), CVD, hypertension, hyperlipidemia, type 2 diabetes, obesity, cancer, COPD, chronic kidney disease, liver disease etiology.
